# Effects of enrofloxacin treatment on the bacterial microbiota of milk from goats with persistent mastitis

**DOI:** 10.1038/s41598-020-61407-2

**Published:** 2020-03-10

**Authors:** Richard Costa Polveiro, Pedro Marcus Pereira Vidigal, Tiago Antônio de Oliveira Mendes, Ricardo Seiti Yamatogi, Magna Coroa Lima, Maria Aparecida Scatamburlo Moreira

**Affiliations:** 1 Laboratory of Bacterial Diseases, Sector of Preventive Veterinary Medicine and Public Health, Veterinary Department, Federal University of Viçosa, Viçosa, MG Brazil; 2 Núcleo de Análise de Biomoléculas (NuBioMol), Center of Biological Sciences, Federal University of Viçosa, Viçosa, MG Brazil; 3 Department of Biochemistry and Molecular Biology, Federal University of Viçosa, Viçosa, MG Brazil

**Keywords:** Classification and taxonomy, Microbiome, Bacterial infection

## Abstract

Antibiotic resistance has become a major concern for human and animal health. As fluoroquinolones have been extensively used in human and veterinary medicine, there has also been the rapid emergence and spread of antimicrobial resistance around the world. Here, we analysed the microbiome of goat milk using samples from healthy goats and those diagnosed with persistent mastitis and treated using the antibiotic enrofloxacin with 16S rRNA amplicon sequencing. We selected a group of 11 goats and 22 samples of milk that did not respond clinically to enrofloxacin treatment. Milk samples were evaluated before and after treatment to verify changes of the microbiota; the three first lactating goats were selected from the healthy control group. The milk samples from the healthy control animals presented a larger abundance of different species of bacteria of the *Staphylococcus* genus, but a smaller number of different genera, which indicated a more specific niche of resident bacteria. The *Firmicutes* phylum was predominantly different between the studied groups. Samples from before-treatment animals had a higher number of new species than those from the control group, and after being treated again. These microbiota received new bacteria, increasing the differences in bacteria even more in relation to the control group. Genotypes such as *Trueperella* and *Mannheimia*, between other genera, had a high abundance in the samples from animals with persistent mastitis. The dysbiosis in this study, with marked evidence of a complex microbiota in activity in cases of the failure of antimicrobial treatment for persistent chronic mastitis, demonstrates a need to improve the accuracy of pathogen identification and increases concern regarding antibiotic treatments in milk production herds.

## Introduction

Antimicrobial therapies have been shown to be increasingly problematic due to the development multiple types of antimicrobial resistance (AMR) mechanisms, and for that reason, therapeutic alternatives to treat multidrug-resistant microorganisms are rapidly dwindling. Fluoroquinolones have been extensively used in human and veterinary medicine as they are considered among the most effective drugs for the treatment of bacterial infections^1,2^.

Enrofloxacin, is a fluoroquinolone exclusively developed for use in veterinary medicine^1,3,4^. This drug is a potent inhibitor of bacterial DNA Topoisomerase II (Gyrase) and the DNA Topoisomerase IV (Topo IV), which are essential enzymes involved in key cellular processes including DNA replication^5–10^. The drug has a broad spectrum of activity, being active against major pathogenic bacteria (both Gram-positive and Gram-negative), mycoplasmas^11^, and also mycobacteria^12^, but is ineffective against obligate anaerobes^13^.

Furthermore, in both mammalian and non-mammalian species, enrofloxacin is partially metabolised in the liver to ciprofloxacin, a primary metabolite of which is cyclopropyl, a potent antimicrobial agent itself^14^. The active substance is characterised by a low host toxicity, being non-mutagenic with a terminal half-life of 2–6 h^1,11,15^. Moreover, of the commonly used antimicrobial agents in mastitis treatment, fluoroquinolones distribute well into an inflamed mammary gland^16–18^ and enrofloxacin has shown a high bioavailability and excellent tissue penetration in goats^15,19,20^.

However, resistance to fluoroquinolones is still occurring at an increasing rate in numerous bacterial species, and their use varies around the world. Therefore, culture-independent techniques are essential to determine the organisms that are present in a given sample and allow for the assessment and utilisation of the genetic wealth they represent. Metagenomics represents a powerful tool to achieve these goals using sequence-based and functional-based approaches^6,21^.

Mastitis, or inflammation of the mammary gland^22,23^, is primarily caused by bacterial intramammary infection (IMI). IMI is the most relevant small ruminant disease and causes severe economic losses to the dairy industry worldwide^24,25^. Several bacterial pathogens can cause IMI, but *Staphylococcus* spp. are the most frequently diagnosed causal microorganisms of IMI in goats and sheep^26^. Other pathogens such as *Streptococcus* spp., *Enterobacteriaceae*, *Pseudomonas aeruginosa*, *Mannheimia haemolytica*, *Corynebacteria* and fungi can produce IMI in small ruminants, but occurrence rates are lower. The high diversity of microorganisms, mainly IMI-causing bacteria, are difficult to treat and control in human and veterinary medicine^26,27^. Dysbiosis, defined as a breakdown in the balance between putative microbial commensals and pathogens, demonstrates that disrupting the microbiota contributes to mastitis pathogenesis and the dissemination of AMR through milk^25,28–31^. Nevertheless, the use of antimicrobials may alter the commensal microbiota of milk that has a protective role of the mammary gland^31,32^.

In human milk and the milk from different ruminants, a mammary gland-specific microbiome has been identified^33–35^. Goat (*Capra hircus*) milk production is of significant importance to the economy in many countries and offers many health benefits^36–40^; this milk has demonstrated high microbial diversity in studies^41,42^ and its consumption has shown potential benefits to intestinal microbiota^43^. Few studies have revealed microbiota in goat milk; however, the main bacterial phyla found are *Proteobacteria*, *Actinobacteria*, *Firmicutes* and *Bacteroidetes* and a variety of genera with *Acinetobacter*, *Agrobacterium*, *Bacteroides*, *Bacillus*, *Enterobacter*, *Massilia*, *Micrococcus*, *Pseudomonas*, *Phyllobacterium*, *Rhodococcus*, *Staphylococcus*, *Stenotrophomonas*, *Stenotrophomonas* and *Streptococcus*^41,42^.

Most cases of IMI are chronic, persistent IMI (PIMI), which are difficult to treat and prone to resurgence, and thus are often accompanied by long-lasting cost-intensive antibiotic treatment and premature culling^22,27,44,45^. Mastitis in small ruminants, especially goats, often persists through the lactation and dry periods, and re-infection is common^46^. PIMI infection can be related to the AMR process, in which the bacterium is capable of evading clearance by antibiotics and the host’s immune system, resulting in long-lasting, persistent infections^44,47^. AMR increases the risk of subsequent microbiome invasion by pathogens, and subsequent disease^48^.

Analyses of microbiota with the use of antibiotics in prophylactic treatments of mastitis in cows^31^, as well as therapeutic treatments^49^, have left doubts as to the efficiency of reducing or eliminating pathogenic bacteria in the milk microbiome. However, knowledge of the microbiota of goat milk-associated pathogens in IMI and the impact of the use of enrofloxacin in cases of PIMI still needs to be determined.

The objectives of this study were to generate knowledge of the microbiome of goat milk using samples from healthy goats and those diagnosed with PIMI after treatment with the antibiotic enrofloxacin, characterised by 16S rRNA amplicon sequencing. Specifically, it was conducted to: (a) describe the microbiome of goat milk from healthy controls, before and after the treatment of IMI; (b) compare the microbial populations of treatment and healthy control groups to evaluate the impact of enrofloxacin on mastitic milk; (c) perform predictive functional profiling of microbial communities and compare the metabolic and functional profiles of the bacteria.

## Results

### Summary of treatments, microbiological tests and sequencing

**Figure 1:**
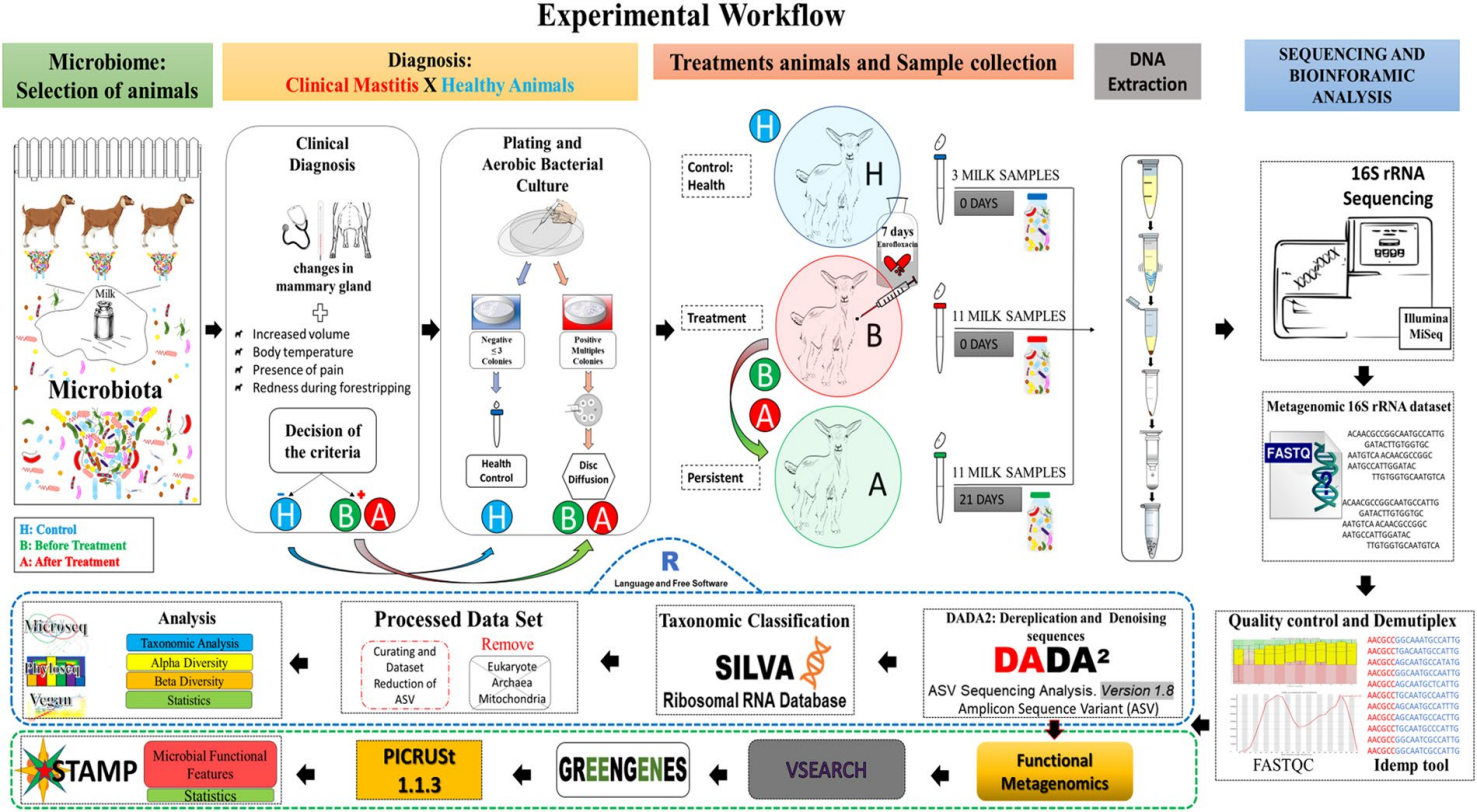
Experimental workflow. Description of the study development process, with the indications of workflow. The letters in blue (H) correspond to healthy controls, (B) to before treatment and (A) to after treatment with enrofloxacin. The black arrows correspond to the workflow until the finalisation of the results.

We selected 25 samples of goat milk, three samples from healthy controls (H), 11 samples from before treatment (B) and 11 samples from after treatment (A) from the same animals, which showed clinical and bacteriological persistence when properly treated for clinical mastitis in another study^50^ with the antibiotic enrofloxacin ^1,4,19,51^. The 22 samples, A and B deeply discerned the microbiological cultures, and were identified as having multiple microorganisms. Three H samples did not show bacteriological growth in the culture medium. Of all 13 tested antibiotics, enrofloxacin was the only antibiotic identified as effective for the treatment mentioned in the methodology (Fig. 1).

The MICs ranged from 0.125 to 16 μg/mL in the isolates prior to treatment (B), whereas in the isolates after treatment (A), the antimicrobial MICs ranged from 0.19 to 16 μg/mL, with only samples M1 and M2 showing 16 μg/mL.

A total of 25 goat milk samples were collected and sequenced in the V4 region of the 16S rRNA gene; quality-filtered reads were demultiplexed and a total of 680,858,000 sequences were used for downstream analyses (mean = 27,234,320 ± SD = 11,734,822 reads/sample). The median length for all reads was 254 bp. In all, 871 taxa identified were used in the analyses.

### Taxonomic classification by different databases and alpha diversity

Four different types of databases were used to conduct the taxonomic classification; amplicon sequence variants (ASVs) that did not meet the purpose of this study were removed, as reported above. Table S1 demonstrates the classification distribution for goat milk in our study, and the Silva database showed the best taxonomic classification (Table S1).

Richness and diversity were analysed to assess whether any divergence was observed across groups. The Chao 1 and Shannon indices were not statistically different between treatment groups (Fig. S1). The results for the rarefaction curves (Fig. S1A) and the paired comparison of alpha diversity (Fig. S1B) did not present statistical significances for alpha diversity and richness, however, there is a possible and apparent difference in richness (Fig. S1A). Thus, when analysing rare curve plots individually (Fig. S1A), the data demonstrate a lower species richness for the H group; six samples increased the richness of group B compared to group A, three decreased and two remained at practically the same level of richness.

### Composition of the bacterial microbiota for each treatment group and the persistent subgroups

**Figure 2:**
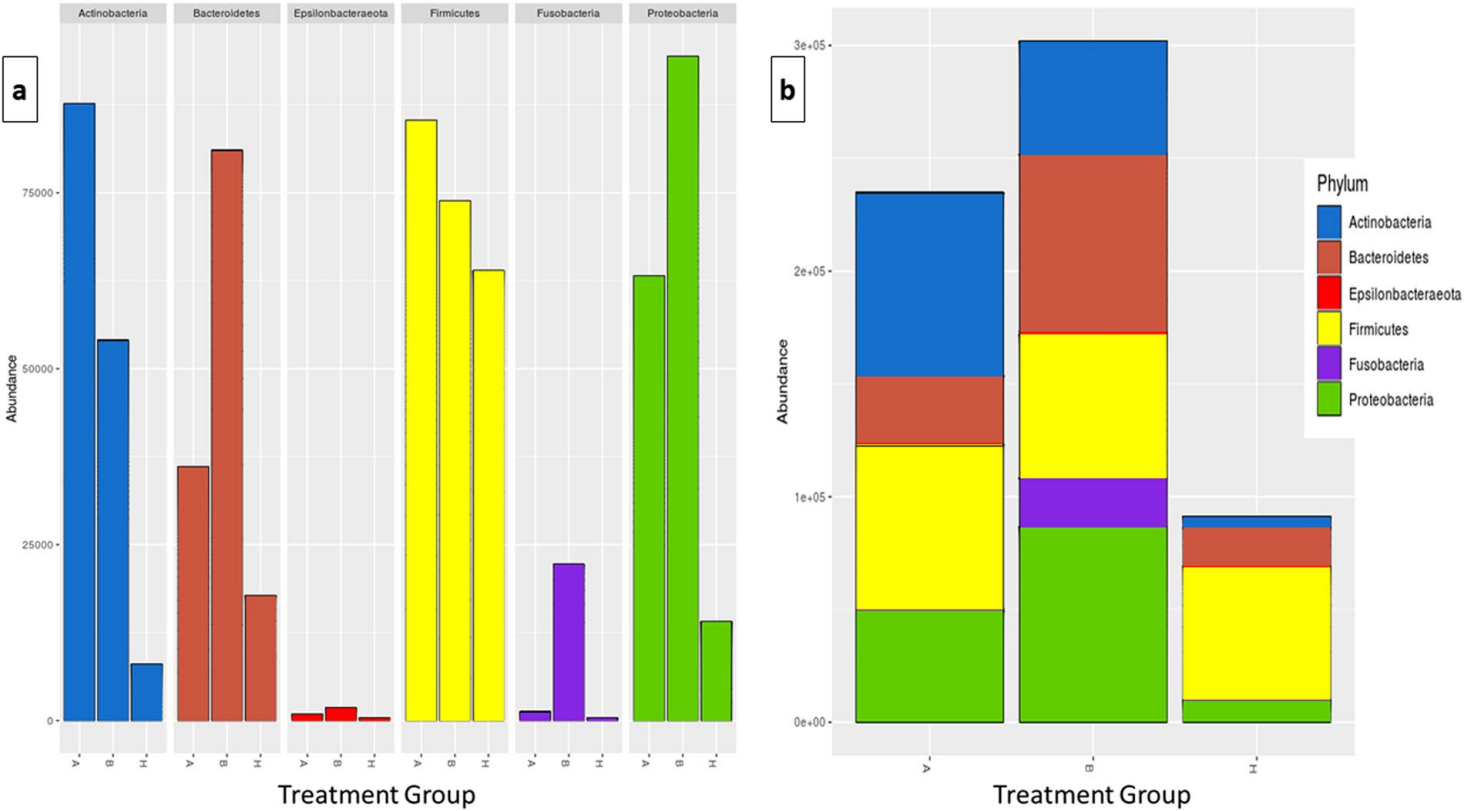
Bacterial microbiota composition, at the phylum level, between treatment groups. (**a**) Taxonomic composition of the phylum and differentially abundant bacterial taxa between the after treatment (A) and before treatment (B) groups and the healthy controls (H), divided at the phylum level, with each colour corresponding to a different phylum. (**b**) Grouped taxonomic composition phylum in terms of the abundance in the treatment group, with each colour corresponding to a different phylum.

The compositions of microbiota, at the phyla level, are shown in Fig. 2. Upon comparison, *Firmicutes* had the highest proportion and presented a significant difference (P = 0.030) in its paired comparison, as shown in Fig. S2 (ANOVA, P < 0.05). Post hoc tests indicated a difference between B and H, P = 0.006. The other phyla, although not showing any significant differences between the taxa, presented different proportions between groups (Fig. 2), such as *Actinobacteria* and *Firmicutes*, which were more abundant in group A, and *Bacteroidetes*, *Fusobacteria* and *Proteobacteria*, which were more abundant in group B.

**Figure 3:**
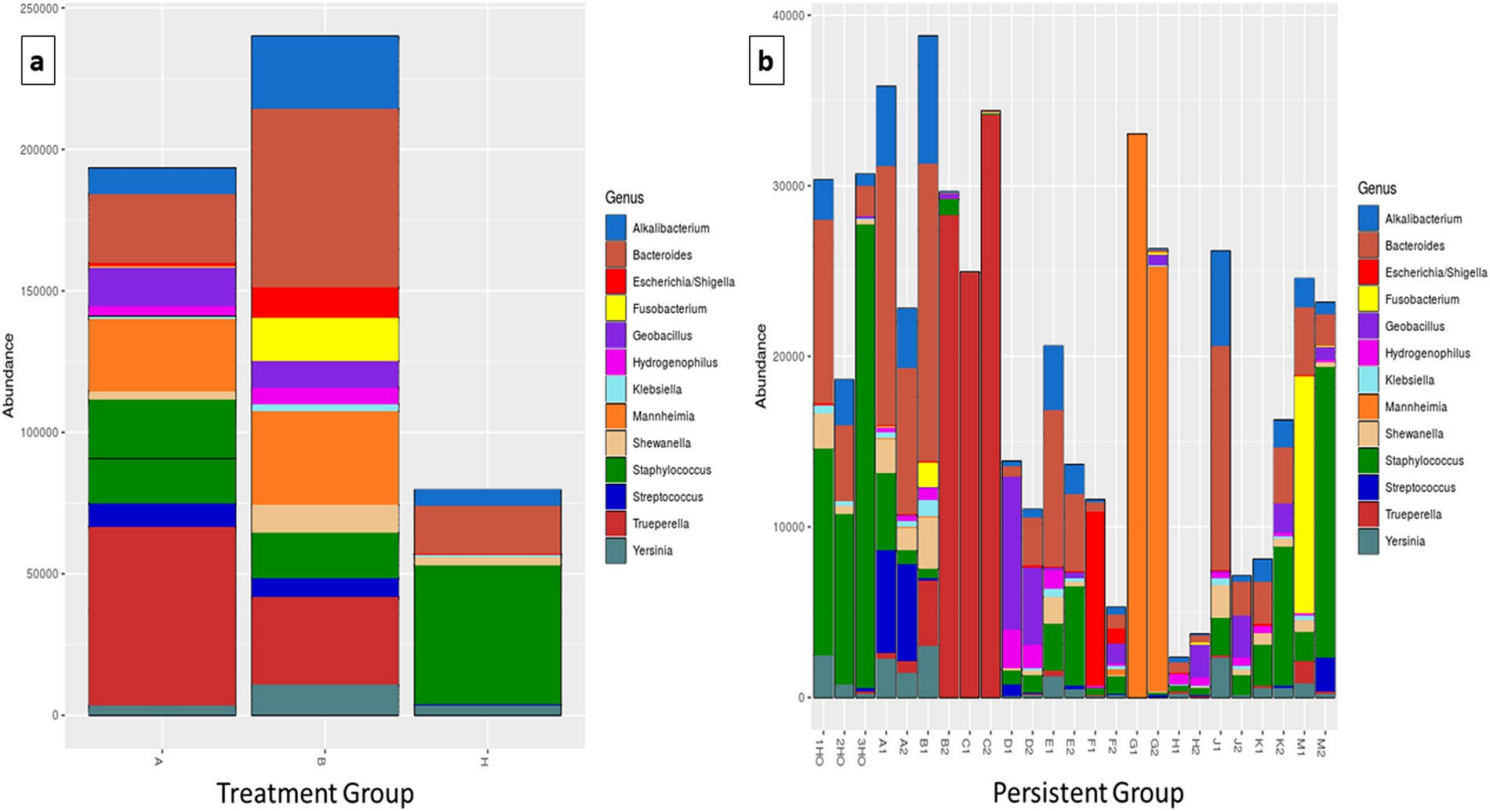
Bacterial microbiota composition, at the genus level, in the group of milk samples prior to treatment (before treatment) and 14 days after treatment (persistent groups): (**a**) Taxonomic composition of genus and differentially abundant bacterial taxa in the 14 days after treatment (A), before treatment (B) and healthy control (H) group, divided at the genus level, with each colour corresponding to a different genus. (**b**) Taxonomic composition of genus and differentially abundant relative bacterial taxa in the different samples of before treatment represented by number (1) and 14 days after treatment represented by number (2). Samples presented as HO are different healthy controls (H) and animals of each group are represented by different letters of the alphabet.

The data exploration, presented by the genera below (Fig. 3a), showed that H had a high abundance of *Staphylococcus* sp., *Bacteroides* sp., *Alkalibacterium* sp., *Shewanella* sp. and *Yersinia* sp. (Fig. 3). The genus *Staphylococcus* sp. showed decreasing relative abundance from H to A and consequently to the B groups. This difference in *Staphylococcus* sp. abundance was significant when H was separately compared with A, and with B, as demonstrated in Fig. S3 (White’s non-parametric t-test, P < 0.05). The major abundant genera between the groups were *Trueperella* sp., *Bacteroides* sp., *Staphylococcus* sp., *Alkalibacterium* sp., *Mannheimia* sp., *Yersinia* sp., *Fusobacterium* sp., *Escherichia* sp./*Shigella* sp., *Streptococcus* sp., *Geobacillus* sp., *Shewanella* sp., *Hydrogenophillis* sp. and *Klebisiellla* sp. (Fig. 3).

Samples B1–B2 showed a significant increase (G-test + Fisher’s, P < 0.01) in abundance of the *Trueperella* sp. genus after enrofloxacin treatment. On the other hand, the genus *Mannheimia* sp. showed a slight but non-significant decrease in abundance, together with appearances of low levels of abundance of several other bacteria in the sample microbiome (Fig. 3b).

In Fig. 2a,b, it can be seen that the abundances of the phyla *Fusobacteria* and genera *Fusobacterium* in group B are higher than those in groups A and H, which shows that antibiotic action may reduce or shift its abundance, directly or indirectly. There are other agents such as *Staphylococcus* sp., occupying the space left in the microbiota, which may have blocked the return to balance of microbiota in a healthy clinical state (HO). Nevertheless, the MIC result shown above for samples M1 and M2 may explain the reduction in *Fusobacterium* spp. and the increase in *Staphylococcus* spp. in Fig. 3b. The technique of classification itself could not distinguish the *Escherichia* sp./*Shigella* sp. genera in this study. These results confirm the complex bacterial aetiology of mastitis in goats.

### Distinction of microbiota by a Venn diagram

**Figure 4:**
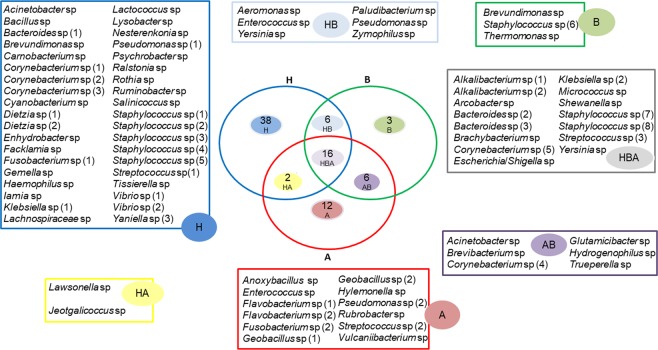
Venn graph with tree groups B, A and H, with the frame distributed for each genus. The treatment groups were equally distributed in different colours, as well as their intercalations, with H corresponding to healthy controls, B to before treatment with enrofloxacin and A to after treatment with enrofloxacin. The frames on the sides of the Venn diagram are identified with the colours of their respective grouping of the central figure of Venn and identify each genus that has been catalogued for their groups. The numbers in front of the genera differentiate one bacteria from the other, since this genre reappears, but with possible different species and subspecies.

The distribution of these genres between groups can best be visualised in a Venn diagram (Fig. 4). In the Venn diagram, all taxa of the bacteria belonging to each treatment group are listed, as well as their intercalations between groups. Some of the assembled genera appear repeatedly in the same groups and in others, due to the occurrence of the gender classification that occurs through the ‘DADA2’ pipeline package, which realises the distinction of sequence variants by as little as one nucleotide, which determines this high distinction best.

The H group had the largest number of bacterial genera (38) distinct from the other groups or in interrelationships; the A group had the second largest number with 12 and the B group had three. The number of genera that were present in the three treatment groups (H, B, A) was 16, while those in H and B were six, B and A were six, and H and A were two genera. Therefore, there were more bacteria from the H group correlated with the B group than from the H group correlated with the A group. In terms of the beta diversity chart (Fig. 4), there was a significant dissimilarity between the H and A samples, which corroborates the distinction that occurred in A with enrofloxacin from H. The genus *Staphylococcus* sp. appeared the most frequently among the groups.

### Differences in microbial composition based on beta diversity

**Figure 5:**
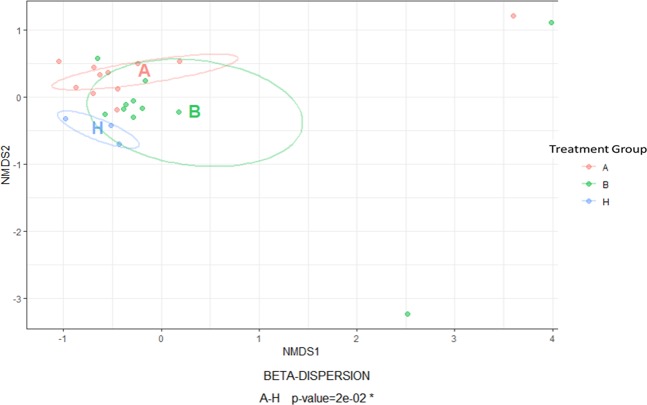
Beta diversity based on Bray–Curtis dissimilarity and non-metric multidimensional scaling. Graphic beta diversity with principle coordinate analysis of the microbial community based on the Bray–Curtis distance between treatment groups in milk microbiota: before treatment (B), after treatment (A) and health control (H), by non-metric multidimensional scaling (NMDS). The position of samples in the NMDS ordination represents the rank order of inter-sample distances, which denotes the similarity between A–B and B–H, and the dissimilarity between A–H (Adonis; R^2^ = 0.10 and P = 0.022).

When comparing B, A and H, the groups that presented significant differences were A–H (Adonis; R^2^ = 0.10 and P = 0.02), which demonstrates that the distribution and abundances of the two groups were different (Fig. 5). Therewith, we could not observe any separation between A–B and B–H, but a pronounced separation between the A–H groups (Fig. 4), which denotes that treatment (A) caused a greater distance from the profile of the resident microbiota (H).

In the beta diversity chart (Fig. 5), the significant dissimilarity between groups H and A contextualises the evidenced results for the same groups in the Venn diagram (Fig. 4), in which we observed only two bacteria in group HA.

### Predicted functional metagenome in personality groups

**Figure 6:**
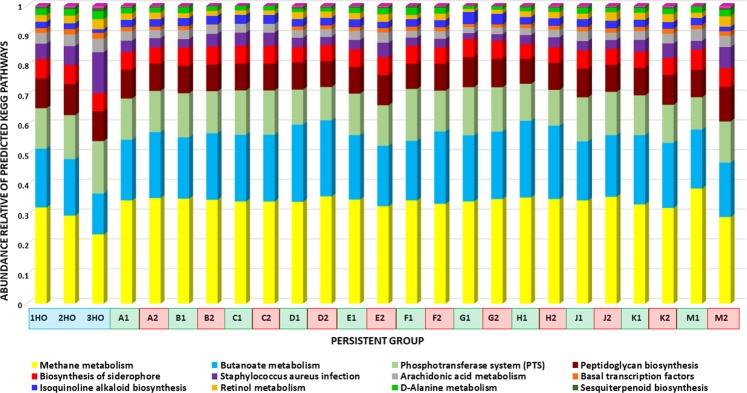
The microbial functional features in different samples of goat milk, belonging to the persistent groups in relative abundance KEGG Level 3. Prediction of the function of the goat milk microbiota from healthy controls (blue), and the persistent groups before treatment (green) and after treatment (red) at KEGG pathways Level 3, transformed into relative abundances. The comparison of the abundance of predicted pathways was performed using the PICRUST and STAMP programs. Significant pathways were selected using an ANOVA (P < 0.05), with a Tukey–Kramer test (0.95).

Next, we analysed the predicted functional pathways in each sample belonging to the persistent group, annotated using the Kyoto Encyclopedia of Genes and Genomes (KEGG) at Level 2 and Level 3 KEGG orthology groups which were significantly different between H, B and A. The composition differences in abundance, in terms of highs and lows between the samples, were 13 traits detected in L3 (Fig. 6) and another four traits detected in L2 (Fig. S4).

KEGG basic features in L2 were energy metabolism, infectious disease, restriction enzyme and transcription-related proteins (Fig. S4). The most prominent and significantly abundant features related to Level 3 analysis were methane metabolism and butanoate metabolism, which demonstrated lower abundance characteristics in the H group in relation to the A and B groups (Fig. S5).

## Discussion

AMR is one of the major public health problems that is rapidly growing around the world^52^ and is accompanied by a decline in the discovery of new antimicrobial agents^53^. The development of AMR raises concerns related to food safety, as it may lead to a decrease in the availability of antibiotics for use in production animals, and thus limit the ability of professionals to control disease in animals raised for the production of food^49^. Our data revealed the diversity and richness of the microbiota in goat milk, contrasting and comparing the main pathogens in a situation of mastitis persistence in a herd of goats treated with the antibiotic enrofloxacin.

Although the groups did not present differences in terms of the indices of alpha diversity, the analysis of the diversity of the microbiota was somewhat complicated, since the interactions between environment, microbiome and host are dynamic, and further, the addition of an antimicrobial will be another distortion factor in this dynamic^54^. In this regard, several studies on antibiotics and alpha diversity have presented different results. For example, in studies of treatments of cows with antibiotics, Shannon diversity and Chao1 richness indices were not significantly different between animals that received an antibiotic (intramammary ceftiofur hydrochloride) and teat sealant or a teat sealant alone^31^. In contrast, another study identified increased alpha diversity in milk microbiota of cows treated with third-generation cephalosporin and experimentally induced mastitis^49^. In mice, it is suggested that antibiotic treatment decreases microbial diversity^55^, and similar results have been obtained in humans^56–58^.

Thus, we first hypothesised that the antibiotic dose and the response of the infection was not sufficient to return to the resident microbiota, although as reported above, enrofloxacin has been shown to be viable in treating mastitis^1,4,19,51^, however the multi-plurality of microorganisms may have aided in the persistence of disease. Second, we hypothesised that healthy animals (H) have more specific microbiota, and during the phase when the animal presents with mastitis, new microorganisms are included that generate an increase in species richness; with the advent of antibiotic treatment and the persistence of mastitis, this microbiota is again altered, but does not return to the specific frame of the healthy phase, and instead ends up adding new microorganisms, causing maintenance of the clinical state of mastitis and an increased imbalance of the microbiota. This microbial imbalance or difference in microbial composition is called dysbiosis^59^. This term is defined as a disturbance of intestinal microbiota homeostasis, or an imbalance in the microbiota itself, due to changes in the functional composition and metabolic activities or changes in the local distribution of resident microorganisms for that particular niche^60,61^. The homeostatic balance of the intestinal microbiota is extremely beneficial to the host^62^, as should occur in the milk of the mammary gland of healthy goats.

The four phyla present here (*Actinobacteria*, *Bacteroidetes*, *Firmicutes*, *Proteobacteria*) are important for goat milk microbiota^41,42^, more specifically the *Firmicutes*, which had significant changes in abundance between the groups (Fig. S2). *Firmicutes* are also an important group of bacteria in cow’s milk^25,31,42,63,64^, but their specific role in the milk microbiome is yet to be determined.

The genus *Staphylococcus* was important in the healthy control group, although in goat milk samples, this genus is more frequently related to mastitis due to IMI in goats and sheep in Brazil^65,66^ and around the world^26^. However, in some studies^66^, this genus stands out in the healthy mammary glands of goats and it has also been reported that such bacteria have been identified in the healthy milk microbiome of humans and cows^25,67,68^. Human breast milk, for example, even if aseptically collected from a healthy woman, can contain *S*. *epidermidis* and *S*. *aureus*^69^.

A study by Jácome *et al*.^70^ noted infection by the genus *Staphylococcus* in primiparous females. Nevertheless, the presence of the genus *Staphylococcus* as native to the indigenous microbiota would not be strange, since the presence of an autochthonous or commensal microbiota is essential for good physiological maintenance and defence against different pathogens in apparently-connected organs, such as the intestine, eyes, ears and urogenital tract in humans^71,72^, as well as the rumen in ruminants and other terrestrial mammals^73,74^. Therefore, as has been observed regarding the microbiota of the intestinal tract of humans, it is suggested that the animal mammary gland also appears to have a specific primary resident microbiota, as noted in this study.

*M*. *haemolytica* and *S*. *aureus* are the main causes of mastitis in small ruminants^75^. The genera *Trueperella* and *Mannheimia* demonstrated almost complete dominance in the samples in which they were present, before and after treatment (Fig. 3), and were likely to be responsible for clinical mastitis^76^. The main genus and species in relation to any mastitis is *M*. *haemolytica*, which causes clinical mastitis, pneumonia and septicaemia in ovines^77,78^. However, reports of subclinical caprine mastitis related to this agent have been found^79^. In Brazil, in the state of Minas Gerais, isolation and identification were carried out of *M*. *haemolytica* bacteria as a causative agent of pneumonia in ovines^80^. Species of the genus *Trueperella* are known to be important opportunistic pathogens in livestock and domestic animals, causing a variety of infections^81,82^, as well as mastitis and uterine infections in cows^83,84^. Mastitis caused by *T*. *pyogenes* is considered of minor importance in goats^22,85^.

The genus *Fusobacterium* was identified with differences in abundance in samples M1 and M2. This genera consists of Gram-negative obligately anaerobic bacteria^86^. However, many fluoroquinolones are considered ineffective against obligately anaerobic bacteria^75^. Thus, the real reason that led to the reduction of the genus *Fusobacterium* and the increase in sequences of *Staphylococcus* spp. in the sample is unclear, but mechanisms using competitive exclusion of microorganisms in microbiomes may have played a role in this reduction^87^. *Fusobacterium* spp. have been identified in high abundance in metagenome studies in milk samples from cows diagnosed with clinical mastitis^25,29^. Metagenomic studies using shotgun sequencing in subclinical mastitis samples have also reported the presence of anaerobic bacteria (*Fusobacteriales* spp., *Bacteroidales* spp.)^88^. *Fusobacterium necrophorum* was detected in high prevalence in mastitis samples in cows and is considered an opportunistic agent; however, the conditions for anaerobic growth of this agent have hindered its isolation in standard isolation methods in culture^25,29^.

Among the factors that can cause a disturbance in abundance^89^, the use of antibiotics, in this case, can disturb the community of species that are commonly less abundant under non-perturbed, equilibrium conditions; these species can become more abundant during or in the wake of disturbance, and can be more easily detected^90^. Furthermore, after disturbance, indigenous community members may die (mortality) or their relative abundances may change^91^. Therefore, following antibiotic treatment, a previously rare microbe may increase in abundance to fill a niche that had been dominated by a microbe with higher antibiotic sensitivity; this can lead to the persistence of the same stable state^89^.

These alterations in the microbiota may indicate that we should change the way mastitis is treated in animal rearing, and that we must take into account custom treatment procedures, as already occurs in human^92^ and animal hospital settings^93^. This type of procedure, using personalised antimicrobial administration, may decrease the possible massive spread of resistance in the dairy industry.

An increase or decrease in methane metabolism, as in this study (Fig. S5), always happens in anaerobic bacteria, including *Bacteroides* spp., which induces its increase or decrease^94^. Moreover, butanoate metabolism plays a critical role in the prevention and treatment of intestinal diseases^95^. In addition, reduced butanoate metabolism and induced methane metabolism are important metabolic processes, as well as the genus *Bacteroides* spp^96^. Thus, we assume the direct influence of the behaviour of the *Bacteroides* genus on the differences of these two metabolic pathways, suggesting that the increase of the genus leads directly to an increase of these pathways, which distinguishes the milk of these diseased animals (B and A) from that of the healthy control (H).

However, care must be taken since fluoroquinolones are also important in human therapeutics^97^. In addition, cross-resistance between enrofloxacin and other antimicrobials of the same class as human ciprofloxacin can occur^98,99^. There is a need for attention to bacterial resistance to enrofloxacin^6,100,101^ and concern about the presence of residues of this drug in human foods^102,103^, in dusts from intensive livestock farming^101^ and the dissemination of livestock waste for wildlife^104^. The use of antimicrobial agents in the food industry can indiscriminately impact human health and presents a high cost to society in terms of AMR; the only way to avoid this is to ensure their judicious use ^6,105^.

## Conclusion

In this study, we demonstrated the dynamics of the microbiota variation that exists in the milk from the goat mammary gland when persistent chronic mastitis (PIMI) occurs after the ineffective treatment with the antibiotic enrofloxacin, using 16S rRNA amplicon sequencing. We demonstrated the high abundance of the genus *Staphylococcus* in samples from healthy animals and hypothesised that it is a common indigenous agent, and then identified the *Firmicutes* phylum as a divisor between the groups. In addition, we demonstrated the predominance of several genera previously known as pathogens of cases of clinical mastitis in goats, dissimilarity between the milk samples from groups of healthy animals and those after treatment, and the metabolic prediction of the importance of methane and butanoate metabolism. More detailed future studies could elucidate which species belong to these genera and the present resistome responsible for mastitis persistence, further detailing the role of the resident microbiota in the face of dysbiosis and the development of AMR.

## Methods

### Ethics statement

The experimental protocol was approved by the Ethics Committee (Comissão de ética no uso de animais – CEUA) of the Federal University of Viçosa, according to protocol number 43/2016. The methods were carried out in accordance with the approved guidelines

### Criteria for selection, treatment of animals and sampling

In this study, only samples from animals belonging to a single herd in the goat sector of the University Federal of Viçosa (Viçosa, Minas Gerais, Brazil), which were diagnosed with PIMI and treated with the antibiotic enrofloxacin, referring to the study carried out by Lima *et al*.^50^, were selected. The healthy control group was selected and the samples were collected at the same time and place. Eleven animals diagnosed with PIMI were selected and three healthy control animals (H) were also selected during the same period.

The animals diagnosed with IMI and PIMI were first evaluated for signs of clinical mastitis and the presence of at least visually abnormal milk (i.e. the presence of flakes, clots, blood or serous milk), as well as changes in the mammary gland, such as an increased volume and body temperature, and the presence of pain and redness during forestripping performed at the milking parlour, in the presence of a veterinarian. In addition, for animals to be considered as having PIMI, they should have presented an individual clinical history with previous appropriate antibiotic treatment, and a clinical status of clinical mastitis.

The pre-requisites for healthy control (H) animals were that the animals presented only first pregnancies or were primiparous, did not present any signs of clinical mastitis during the physical examination or a history of mastitis. None of these goats had any clinical history of any intervention or treatment in which intramammary, injectable, oral antibiotics or other medicinal products that could have interfered with the results of our analyses were used. In addition, only goats with a negative bacterial culture were selected.

All samples that were harvested from each goat, from both quarters separately, and harvested prior to treatment on day 1 were named before treatment (B). Goats assigned to the treatment of IMI received daily and intramuscular infusions containing enrofloxacin (Kinetomax®, Bayer S.A., Brazil) at a dose of 5 mg/kg every 24 hours for seven days. The animals were re-examined 21 days after starting treatment, and the samples were harvested and thus named after treatment (A) and the goats were diagnosed with PIMI, whereas those in the control group (H) had their medical condition tracked by veterinarians^49^. Test results by the Kirby–Bauer disc diffusion method were the basis for the decision to choose enrofloxacin treatment.

Milk samples were collected by a trained veterinarian member of the research team following the standard recommendations of the National Mastitis Council’s Laboratory Handbook on Bovine Mastitis^106^. For goats in the PIMI-treated group (A), sampling was performed after milking of the untreated quarter and the milk sampled immediately before the intramuscular infusion treatments were applied. For goats in the IMI group (B), sampling was performed in a quarter with clinical signs of IMI and identified as the same that would be collected in the PIMI group (B). Approximately 15 mL of milk was collected before milking and after the external cleaning of the ceiling with alcohol 70 (ethyl alcohol hydrate 70° INPM), with the first jets of milk discarded and the teats of the animal individually dried with paper towels. Samples were immediately refrigerated at 4–7 °C, transported to the laboratory on ice and processed for microbiological tests within six hours.

### Bacteriological examination and antibiotic sensitivity test

For the isolation of the bacteria, 100 μL samples of pre-homogenised milk were used for full aerobic bacteriological culture and were spread on Columbia Agar supplemented with 5% sheep blood. All milk samples were directly cultured for aerobic bacteria using described standard culture techniques^107–109^. Plates were read after 24, 48 and 72 hours. The plates having more than three colonies after 48 to 72 h incubation at 37 °C were considered positive from an individual milk sample; for H samples, plates that did not demonstrate bacterial development were considered negative^108,110^. Additionally, a 2 mL milk aliquot was stored at −80 °C until further DNA extraction.

The antimicrobial susceptibility tests performed in this study were previously described by Lima *et al*.^50^. Briefly, antibiotic resistance testing was done by the Kirby–Bauer disc diffusion method, following the recommendations of the Clinical and Laboratory Standards Institute^111^. Bacterial colonies were inoculated into brain heart infusion agar (Oxoid, UK) and incubated. Thirteen antimicrobials that are normally prescribed in the treatment of mastitis (ampicillin, neomycin, oxacillin, penicillin G, enrofloxacin, ciprofloxacin, gentamicin, ceftiofur, sulphadiazine + trimethoprim, cephalexin, cefepime and cefaperazone) were tested. Minimal inhibitory concentrations (MICs) for enrofloxacin were performed using the Etest method (BioMérieux, Marcy l’Étoile, France). However, due to acute mastitis and the need for rapid treatment, only after completion of seven days of enrofloxacin treatment were these MIC results available.

### DNA extraction, amplification of the 16S rRNA gene and sequencing

The total DNA of the milk samples was extracted using the QIAmp DNA kit min (QIAGEN, Valencia, CA), following the process protocol ‘Blood or Body Fluid Spin Protocol’ (Spin Protocol), with modifications described by Kuehn *et al*.^64^. The concentration and purity of the DNA were quantified by spectroscopy (optical density) on a NanoDrop® Therm Fisher Scientific Spectrophotometer (Waltham, Massachusetts, USA)^29^. Samples of extracted DNA were sent to the Argonne National Laboratory (Lemont, IL, USA) in an ice-dry isothermal box at −78 °C, duly identified, for sequencing.

In the Argonne Laboratory (Argonne, IL, USA), the V4 hypervariable region of the bacterial 16S rRNA gene was amplified from genomic DNA by Polymerase chain reaction using the primers 515 F and 806 R optimised for the Illumina MiSeq platform (Illumina Inc., San Diego, CA)^112^, with MiSeq Reagent Kit V2.

### Sequence and bioinformatics analyses

The sequences were demultiplexed using the ‘Idemp’ program (https://github.com/yhwu/idemp). The package ‘DADA2’ pipeline (version 1.8) in R^113^ was used to infer the ASVs present in each sample^114^. ASV methods have demonstrated sensitivity and specificity that is as good or better than operational taxonomic units (OTUs), identifying the distinction of sequence variants by as little as one nucleotide^115^. Bioinformatics processing largely followed the DADA2 tutorial (https://benjjneb.github.io/dada2/tutorial.html). Forward and reverse read pairs were trimmed and filtered, truncated at 150 nt and reverse read at 150 nt, with up to two bases of ambiguous errors allowed, and each read was required to have less than two expected errors based on their quality scores. ASVs were independently inferred from the forward and reverse of each sample using the run-specific error rates, and then read pairs were merged. Chimeras were identified for each sample and removed if identified in a sufficient fraction of the samples by method consensus. Taxonomic assignment was performed against the Silva v132 database, using the implementation of the Ribosomal Database Project (RDP) Classifier, a naïve Bayesian classifier, available in the package ‘DADA2’ in R in default parameters^116,117^. We also performed classification with the databases Greengenes version (13_8)^118^, HITdb v1.00^119^ and rdp_train_set_14^120^ for the purposes of comparison. However, the Silva database best classified up to the genus level.

Next, using the R^113^ package ‘phyloseq’^121^, we removed any ASVs without a bacterial phylum assignment, assigned as *Archaea*, chloroplast or mitochondrial origin. To simplify downstream analyses and to reduce the noise of the analyses, we applied a prevalence and abundance threshold for bacterial ASVs, in which taxa were kept only if they were found at a minimum frequency of 100 in at least one sample. We did not perform non-rarefied data due to the characteristics of our data^122–124^. The alpha diversity indices were explored by analysis of variance (ANOVA) test and analyses of beta dispersion; the Bray–Curtis dissimilarities were calculated for each group by performing non-metric multidimensional scaling (NMDS)^125^ using the package ‘microbiomeSeq’ (https://github.com/umerijaz/microbiomeSeq.git) in R^113^. This NMDS was subjected to PERMANOVA^126^ and 999 permutations. A Venn diagram was constructed from the previously filtered table of ASVs with certain constraints for grouping, so that a particular genus was grouped if it was identified more than six times in groups B, A or H.

Abundance differences between groups H, B and A were compared in the statistical analysis of metagenomic profiles (STAMP)^127^ using a one-way analysis of variance (ANOVA)^128^, followed by a Tukey–Kramer test with a post hoc test (0.95) and the Benjamini–Hochberg procedure to control for false discovery rate (FDR)^129^. A two-sided G-test (w/Yates’)^130^ and Fisher’s exact test^131^ was implemented in STAMP for statistical analysis of two samples^132^. The H control group was compared to groups B and A via White’s non-parametric t-test^133^ using a bootstrap of 0.95. All statistical analyses were performed with statistical significance accepted when P < 0.05, except for the G-test implemented with P < 0.01.

### Predictive functional profiling of microbial communities

The software PICRUSt^134^ was used to predict the functional gene content of metagenomic samples based on raw 16S rRNA marker gene sequences from the DADA2 output files. The analysis was conducted on 97% similarity-clustered OTUs as picked using VSEARCH^135^ in packages R^113^ since PICRUSt requires closed-reference OTU picking using the Greengenes database. OTUs were normalised by copy number and a new matrix of predicted functional categories was created using KEGG database. The STAMP^127^ software package was used to analyse the predicted metagenomic function of the communities; in this way, the differentially abundant KEGG pathways at Levels 2 and 3 were compared. The three groups (H, B, A) were compared using an ANOVA followed by a Tukey–Kramer post hoc test (0.95) with statistical significance accepted when P < 0.05.

## Supplementary information

Supplementary information.

## Data Availability

The DNA sequences generated and analysed during the current study are available in the NCBI SRA repository under BioProject PRJNA575577. Other data from the study are available from the corresponding author upon reasonable request.
