# Supplementary material for: Effects of enrofloxacin treatment on the bacterial microbiota of milk from goats with persistent mastitis
Source: Sci Rep. 2020 Mar 10;10:4421. doi: 10.1038/s41598-020-61407-2 (PMC7064484; doi:10.1038/s41598-020-61407-2)
Supplement: Supplementary file 1 — Supplementary information. [file 41598_2020_61407_MOESM1_ESM.pdf]

Effects of enrofloxacin treatment on the bacterial microbiota of milk from goats with persistent mastitis

Richard Costa Polveiro[1], Pedro Marcus Pereira Vidigal[2], Tiago Antônio de Oliveira Mendes[3], Ricardo Seiti Yamatogi[1], Magna Coroa Lima[1], Maria Aparecida Scatamburlo Moreira[1*].

[1] Laboratory of Bacterial Diseases, Sector of Preventive Veterinary Medicine and Public Health, Veterinary Department, Federal University of Viçosa, Viçosa, MG, Brazil.

[2] Núcleo de Análise de Biomoléculas (NuBioMol), Center of Biological Sciences, Federal University of Viçosa, Viçosa, MG, Brazil.

[3] Department of Biochemistry and Molecular Biology, Federal University of Viçosa, Viçosa, MG, Brazil.

*E-mail: masm@ufv.br

Supplementary table and figures legends:

Supplementary Table S1: Indicative table of best taxonomic attribution of ASVs. Indicative table of best taxonomic attribution of ASVs.

Supplementary Fig. S1: A. Rarefaction curve; B. Box plots of pairwise comparison of alpha diversity of the goat milk microbiota bacteria in treatment groups; C. Table with the Diversity Alpha indices corresponding to the Observed ASVs, Chao1 and Shannon.

Supplementary Fig. S2: Box plots of pairwise comparison between abundance of ASVs of Filo *Firmicutes*.

Supplementary Fig. S3. Difference abundance of different bacterial genera between treatment groups.

Supplementary Fig. S4. The microbial functional features in goat milk samples in A (after -treatment), B (before-treatment) and H (healthy control) groups demonstrate relative abundance KEGG level 2.

Supplementary Fig. S5. Prediction of the function of butanoate and methane metabolism of the goat's milk microbiota from healthy - controls (blue), and persistent before - treatment (green) and after - treatment (red) at KEGG level 3.

Supplementary Table S1.

**Databases**	Kingdom	Phylum	Class	Order	Family	Genus	Species
**Silva**	99,01%	97,42%	96,76%	93,91%	86,81%	67,37%	6,65%
**Greengenes**	99,79%	98,67%	97,19%	93,54%	80,55%	52,80%	11,19%
**RDP**	99,08%	93,81%	89,46%	85,38%	76,76%	60,21%	6,03%
**HITDB**	NA	91,05%	83,58%	79,60%	68,37%	54,00%	22,42%

Indicative table of best taxonomic attribution of ASVs. Percentage of identification of goat milk at taxonomic levels by different databases. Classification performed with the databases Silva v132, Greengenes version (13_8), HITdb v1.00 and rdp_train_set_14.

Supplementary Fig. S1.

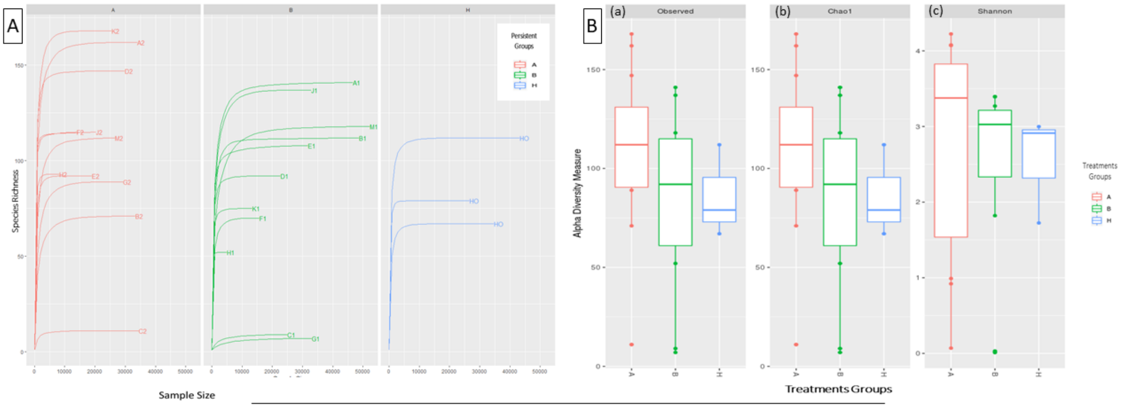

C

Persistent Groups	Treatments Groups	Chao1	Shannon	Persistent Groups	Treatments Groups	Chao1	Shannon
**1HO**	HO	112	2.99836594424563	**F1**	B	70	1.82131768103088
**2HO**	HO	79	2.91234233447382	**F2**	A	115	4.07128310071534
**3HO**	HO	67	1.72469642229424	**G1**	B	7	0.01248645040506
**A1**	B	141	3.02713551656082	**G2**	A	89	0.99064404295691
**A2**	A	162	3.37673172024221	**H1**	B	52	3.27053856227657
**B1**	B	112	2.94622639349989	**H2**	A	93	3.5806471863018
**B2**	A	71	0.920562253381132	**J1**	B	137	2.84405724379255
**C1**	B	9	0.029750813190768	**J2**	A	115	4.22338054013929
**C2**	A	11	0.069602632411666	**K1**	B	75	3.39222282019528
**D1**	B	92	3.15788403432187	**K2**	A	168	3.39764166627452
**D2**	A	147	4.07755875856296	**M1**	B	118	3.03271644552321
**E1**	B	108	3.39578562760161	**M2**	A	112	2.08465881723363
**E2**	A	92	3.0629429036361				

Rare curves, alpha diversity and table with indices alpha diversity. **A**. Rare Curves. **B**. Alpha diversity: (**a**) Observed OTU richness, (**b**) Estimated taxonomic richness (Chao 1), (**c**) taxonomic diversity (Shannon index). **C**. Table with the indexes of alpha diversity.

Supplementary Fig. S2.

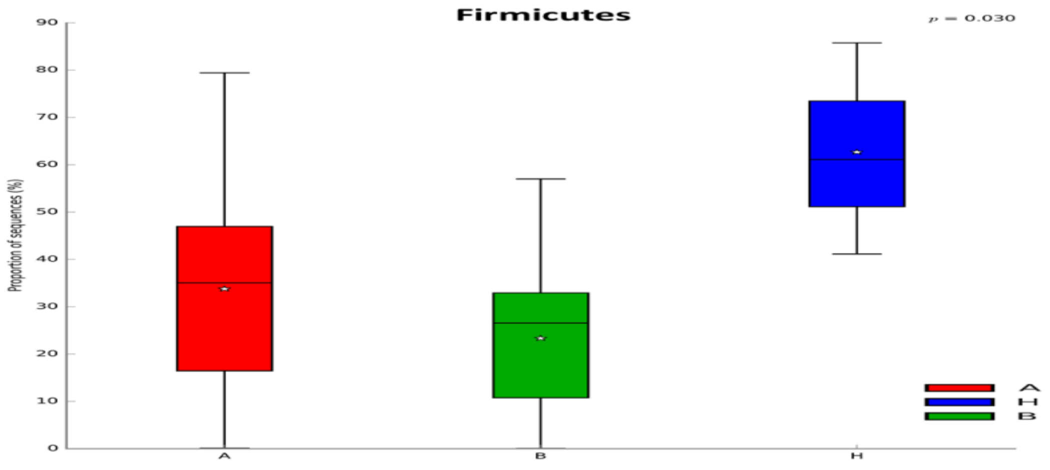

Box plots of pairwise comparison between abundance of ASVs of Filo *Firmicutes*. The color represents the proportion of abundance for each group related to the Phylum *Firmicutes*, A (after treatment), H (healthy-controls), B (before treatment). The markings (stars) on the box indicate significant differences between treatments that were p = 0.030. The tests were performed with ANOVA of P <0.05, Post-hoc test turkey-kramer, 0.95)

Supplementary Fig. S3.

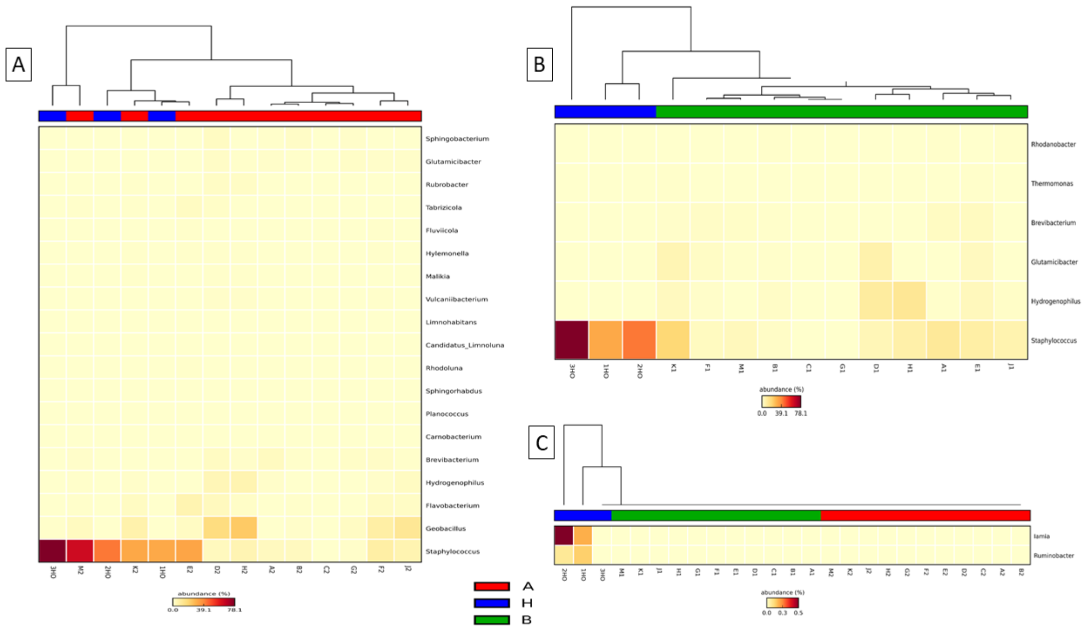

Difference abundance of different bacterial genera between treatment groups. **A.** Differential abundance between groups A-H (Statistics White's non-parametric t-test, P <0.05). **B.** Differential abundance between groups H-B (Statistics White's non-

parametric t-test, P <0.05)**.** **C.** Differential abundance between all groups (before treatment(B), After treatment (A), health-control (H) with significance for the genus *Iamia* sp of p = 0.04 and the genus *Ruminobacter* sp of p = 0.02, statistic ANOVA test (P <0.05), Tukey-Kramer (0.95)**.** The farthest neighbor was the method for calculating distances between clusters in hierarchical clustering. All tests were performed in the STAMP program.

Supplementary Fig. S4.

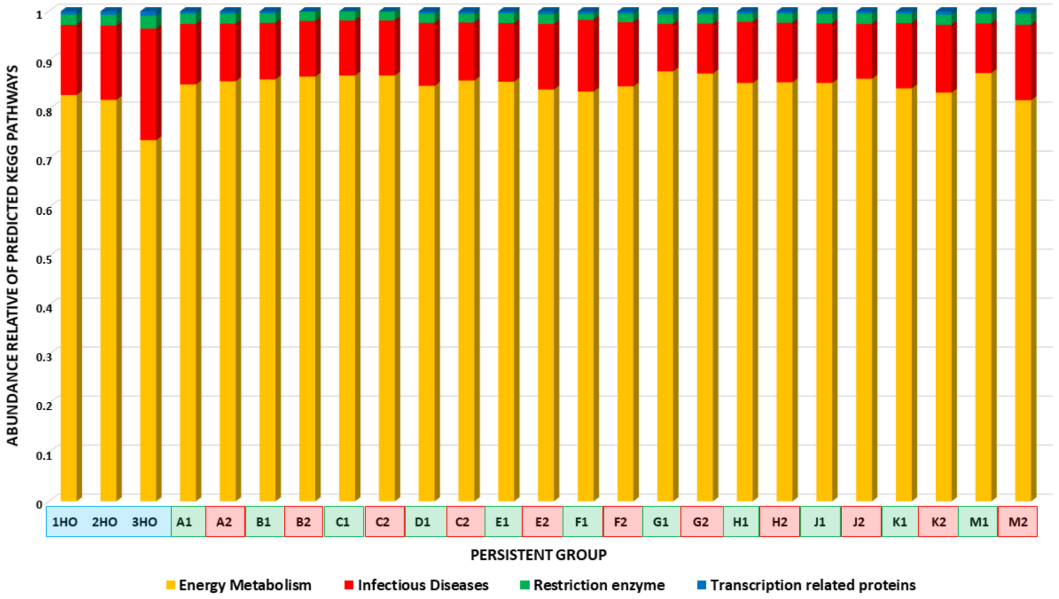

The microbial functional features in goat milk samples in A (after -treatment), B (before-treatment) and H (healthy control) groups demonstrate relative abundance KEGG level 2. Function prediction were transformed into relative abundance. In samples presented as HO, are healthy controls (H) and the others represent by number 1 - before-treatment (B) and by number 2 – after treatment (A), which are arranged as: A1-A2, B1-B2 until M1-M2. The comparison of the abundance of predicted KEGG pathways was performed by the PICRUST and STAMP programs. The significant pathways were selected by ANOVA (P <0.05), with post-hoc test tukey kramer (0.95).

Samples presented as HO are healthy controls (H) and the others represent by number 1 - before-treatment (B) and by number 2 – after treatment (A), which are arranged as: A1-A2, B1-B2 until M1-M2.

Supplementary Fig. S5.

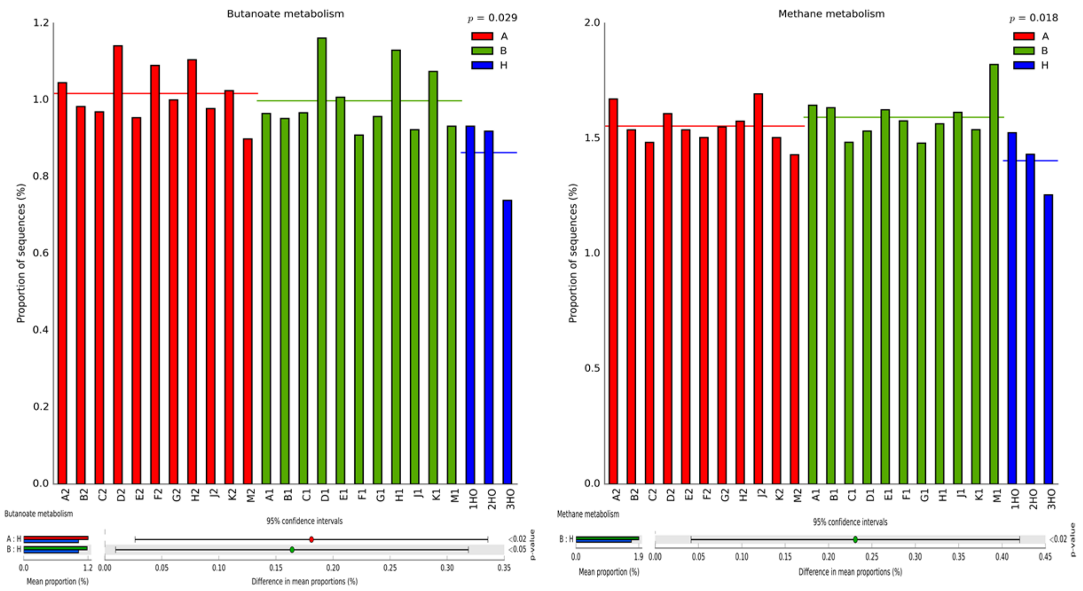

Prediction of the function of butanoate and methane metabolism of the goat's milk microbiota from healthy - controls (blue), and persistent before - treatment (green) and after - treatment (red) at KEGG level 3. The comparison of the abundance predicted with the demonstration of the microbial functions of butanoate and methane metabolism by KEGG in the level 3 category, being averaged over each sample from the persistent group. The values below demonstrate the proportion of means and difference between the proportions of the mean in percentages. KEGG pathways was performed by the PICRUST and STAMP program. The significant pathways were selected by ANOVA (P <0.05), with post-hoc test tukey kramer (0.95).
